# Atopic dermatitis in childhood and pubertal development: A nationwide cohort study

**DOI:** 10.1016/j.jdin.2024.09.018

**Published:** 2024-11-22

**Authors:** Camilla Lomholt Kjersgaard, Andreas Ernst, Pernille Jul Clemmensen, Lea Lykke Harrits Lunddorf, Linn Håkonsen Arendt, Nis Brix, Onyebuchi A. Arah, Mette Deleuran, Cecilia Høst Ramlau-Hansen

**Affiliations:** a Department of Public Health, Research Unit for Epidemiology, Aarhus University, Aarhus, Denmark; b Department of Urology, Aarhus University Hospital, Aarhus, Denmark; c Department of Obstetrics and Gynecology, Aarhus University Hospital, Aarhus, Denmark; d Department of Clinical Genetics, Aarhus University Hospital, Aarhus, Denmark; e Department of Epidemiology, Fielding School of Public Health, University of California, Los Angeles (UCLA), Los Angeles, California; f Department of Statistics and Data Science, UCLA, Los Angeles, California; g Practical Causal Inference Lab, UCLA, Los Angeles, California; h Department of Dermatology, Aarhus University Hospital, Aarhus, Denmark

**Keywords:** atopic dermatitis, atopy, cohort study, eczema, epidemiology, menarche, pubertal development, pubertal milestones, puberty, Tanner stages

## Abstract

**Background:**

Atopic dermatitis (AD) might delay puberty, but research is lacking.

**Objective:**

To investigate the association between AD and puberty.

**Methods:**

A subcohort within the Danish National Birth Cohort includes children born between 2000 and 2003, with mothers reporting doctor-diagnosed AD at 6 months, 18 months, and 7 years old. The National Patient Registry identified hospital-diagnosed AD. From 11 years, the children give half-yearly information on pubertal development. We estimated the mean age difference in months at attaining Tanner stages 1 to 5 and the development of axillary hair, acne, first ejaculation, voice break, and age at menarche, using an interval-censored regression model.

**Results:**

In total, 15,534 children participated, 21.5% had self-reported doctor-diagnosed AD and 0.7% had hospital-diagnosed AD. For girls with self-reported doctor-diagnosed AD, the average age difference at reaching all pubertal milestones was 0.0 months (95% confidence interval [CI]: −0.8; 0.8), and for hospital-diagnosed AD, it was −0.3 months (95% CI: −5.4; 4.8). For boys, the average age difference was 0.1 months (95% CI: −0.6; 0.9) and −0.3 months (95% CI: −3.6; 3.0), respectively.

**Limitations:**

No information on treatment was available. Missing data on covariates (<5%) were not addressed.

**Conclusion:**

No association was found between AD and puberty in either girls or boys.

**Capsule Summary**

• Previous studies on atopic dermatitis and puberty are limited, some suggest a link between atopic dermatitis and delayed puberty, akin to other chronic inflammatory diseases in childhood.
• In this study, atopic dermatitis does not seem to affect pubertal development, which is reassuring for young patients entering puberty and their future reproductive health.

## Introduction

Childhood atopic dermatitis (AD, synonym: atopic eczema or eczema) may influence puberty onset through several mechanisms, such as complex immunological and hormonal interactions,^1^^,^^2^ potentially delaying pubertal development. Altered pubertal development is associated with poor overall and reproductive health in adult life.^3^, ^4^, ^5^, ^6^, ^7^

AD is an inflammatory skin disease^8^ and among the most frequent chronic diseases in childhood and adolescence, affecting up to 15% to 20%^9^ of children and 2% to 10% of adults.^8^ It is closely linked to asthma,^10^ hay fever, and food allergies.^11^^,^^12^ Asthma and asthma treatment along with other chronic inflammatory childhood diseases have been associated with later pubertal timing and delayed physical development.^1^^,^^2^^,^^13^, ^14^, ^15^, ^16^, ^17^, ^18^

Only one study has investigated AD and puberty; however, it was limited to girls and reported only a combined estimate for AD, asthma, and allergies, observing an earlier pubertal development.^19^ The majority of existing literature on the potential consequences of AD focuses on childhood physical development, such as height, with inconsistent results.^20^, ^21^, ^22^ These studies suggest that impaired childhood physical development may be temporary,^17^^,^^23^, ^24^, ^25^, ^26^, ^27^ raising the hypothesis that this temporary impairment might be due to delayed onset of the adolescent growth spurt and, consequently puberty.

AD is hypothesized to delay pubertal development through a compromised skin barrier function. This increases the absorption of endocrine-disrupting chemicals and allergens and allows the penetration of larger compounds than normal skin.^28^^,^^29^ Other potential mechanisms include sleep disturbances, resulting in disturbances of the hormone balance,^30^^,^^31^ a restricted diet because of food allergies,^32^^,^^33^ treatment for example topical glucocorticoids,^17^^,^^34^^,^^35^ or the overactive immune system itself may be involved.^1^

We aimed to investigate the association between AD and pubertal development. We hypothesized that childhood AD is associated with later pubertal development.

## Methods

### Study population

The present study is based on information from the population-based cohort, the Danish National Birth Cohort (DNBC),^36^^,^^37^ and its subcohort, the Puberty Cohort.^38^ From 1996 to 2002, more than 100,000 pregnancies (participation rate: 60%) were recruited to the DNBC. The information on demographic, health, and lifestyle factors were collected by computer-assisted interviews twice during pregnancy and when the children were 6 months old (response rate of 69%) and 18 months old (response rate of 65%). The children were also followed up with questionnaires when they were 7 years old (response rate of 63%) and 11 years old (response rate of 55%).^38^

**Fig 1:**
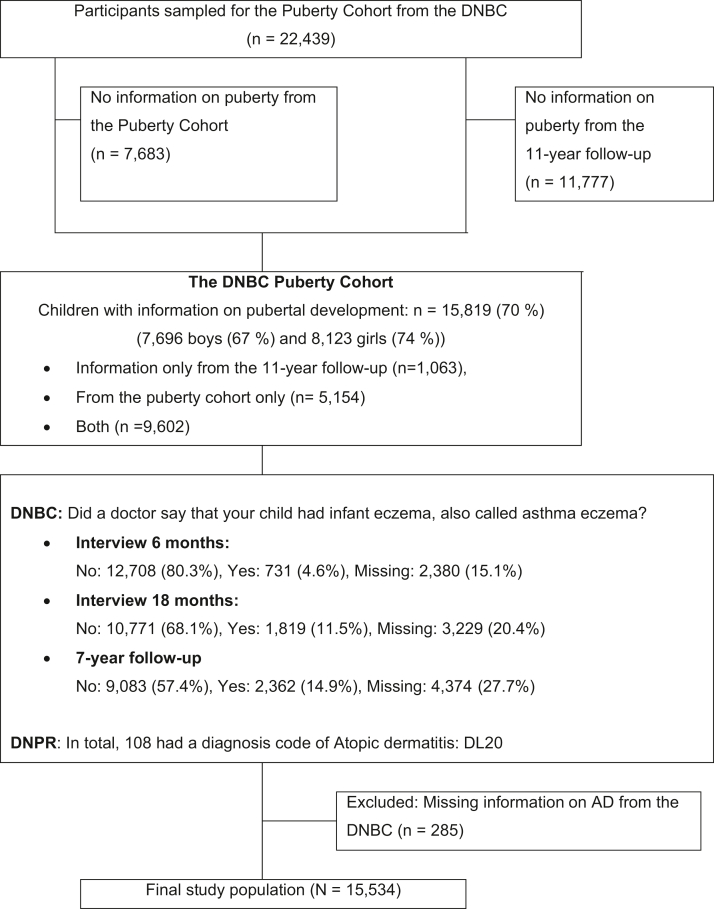
Flow diagram of participants according to atopic dermatitis in childhood and pubertal development, in the Puberty Cohort, Danish National Birth Cohort, Denmark, 2012-2021. *AD*, Atopic dermatitis; *DNBC*, Danish National Birth Cohort; *DNPR*, Danish National Patient Registry.

In 2012, the Puberty Cohort was established and included live-born singletons born between 2000 and 2003 by mothers from the DNBC. Children born by mothers, who replied to the first questionnaire in the DNBC and had not withdrawn by May 2012, were eligible for participation (*n* = 56,641). The children were sampled for invitation according to 15 prenatal and perinatal exposures thought to be of interest for the timing of pubertal development (28 sampling frames). Additionally, a random sample of 8000 children was collected. The sampling has been described in more detail elsewhere.^39^ In total, 22,439 children were invited half-yearly from the age of 11.5 years to provide information on their current pubertal development until full maturity or turning 18 years of age, whichever came first. In all, 14,756 children returned at least one questionnaire. Additionally, 10,688 children answered similar pubertal questions in the 11-year follow-up in the DNBC. When combining the 2 data sources, we had information on 15,819 children (7696 boys and 8123 girls) which corresponds to 70% of the invited population. In total, 98,195 questionnaires (median: 6 questionnaires, range 1-15) were completed (see Fig 1).

### Exposure assessment—AD

The mother gave information on childhood AD when the child was 6 months, 18 months, and 7 years old. Based on responses to questions regarding AD, we categorized participants’ exposures as follows: (1) no AD, (2) self-reported, doctor-diagnosed AD, and (3) hospital-diagnosed AD. In total, 51.7% gave information about AD in all questionnaires throughout childhood (Fig 1). We excluded children lacking information on AD from the DNBC (*n* = 285).

Children had self-reported doctor-diagnosed AD if the mother reported that a doctor (typically their own general practitioner) had diagnosed the child with AD when the child was 6 months, 18 months, or 7 years old. Children had hospital-diagnosed AD if they received an AD diagnosis before the age of 8 years, according to the International Classification of Diseases 10th revision: DL20 from the Danish National Patient Registry (DNPR).^40^ The diagnosis had to be the primary reason for hospital contact. Mainly severe cases of AD are referred to a hospital in Denmark and, thus, registered in the DNPR.^41^

In a sensitivity analysis, we used a diagnostic algorithm by Benn et al,^42^ a combination of questions from the DNBC was used to classify AD when the child was 18 months old. This was not used as the primary analysis since more than 3000 children had missing information on the exposure as some questions were not included in version 1 of the questionnaire in DNBC and not all children have had the diagnosis at the age of 18 months.

In a subanalysis, we investigated persistent or recurrent AD when the child was 7 years old and pubertal development. Persistent AD was defined as active AD at 7 years in the AD group: yes/no, and the rash had to be located at AD-typical places with activity within the last 12 months.

### Outcome assessment—pubertal development

Information on pubertal development was collected through web-based questionnaires, with illustrations and explanatory texts on the current Tanner stage (stages 1 to 5 on pubic hair development, breast development, and genital growth). Furthermore, the participants were asked to state whether they had had their first ejaculation (if yes, which year and month), menarche (if yes, which year and month), voice break, axillary hair growth, and acne.

### Covariates

Based on the literature, directed acyclic graphs were used a priori to identify potential confounders and mediators (Supplementary Fig 1, available via Mendeley at https://data.mendeley.com/datasets/4jhwhhnt93/2). We included the following potential confounders in the analyses (Table I): Highest socioeconomic status of parents, cohabitation of parents during pregnancy, maternal age at menarche, maternal prepregnancy body mass index, and maternal smoking during pregnancy. We had information on the maternal lifestyle, health, and social class of parents from the interviews in the DNBC. The covariates are categorized as shown in Table I.

**Table I:** Maternal and child characteristics according to child atopic dermatitis among 15,534 boys and girls in the Puberty Cohort, Danish National Birth Cohort, Denmark, 2012-2021

Baseline characteristics	Atopic dermatitis			Missings
	No (%)	Self-reported doctor-diagnosed (%)	Hospital-diagnosed (%)	
	*n* = 12,085 (77.8)	*n* = 3341 (21.5)	*n* = 108 (0.7)	
Child characteristics, *n* (%)				
Gender				-
Girl: 7972 (51.3)	6297 (52.1)	1630 (48.8)	45 (41.7)	
Boy: 7562 (48.7)	5788 (47.9)	1711 (51.2)	63 (58.3)	
Birth weight, mean (SD)				0.4%
Girl	3469.7 (568.0)	3495.4 (553.7)	3472.7 (479.7)	
Boys	3584.1 (619.8)	3609.9 (597.9)	3540.0 (659.8)	
BMI child, mean (SD)†				30.3%
Girls	15.6 (1.8)	15.6 (1.7)	16.2 (2.2)	
Boys	15.7 (1.7)	15.7 (1.7)	15.9 (1.6)	
Activity in AD at 7 y				26.4%
Yes	-	736 (26.0)	47 (55.3)	
No	8526 (100)	2092 (74.0)	38 (44.7)	
Benn et al algorithm for AD at 18 mo				18.3%
Yes	416 (4.3)	1677 (58.5)	67 (74.4)	
No	9323 (95.7)	1188 (41.5)	23 (25.6)	
Maternal characteristics, *n* (%)				
Maternal atopy (AD, asthma, hay fever, and/or food allergy)‡				0.2%
Yes	2654 (22.0)	1010 (30.3)	30 (28.0)	
No	9414 (78.0)	2323 (69.7)	78 (72.0)	
Highest socioeconomic status of the parents				0.2%
High grade professional	2791 (23.1)	803 (24.1)	28 (26.2)	
Low grade professional	3947 (32.7)	1160 (34.8)	28 (26.2)	
Skilled worker	3389 (28.1)	852 (25.5)	31 (28.0)	
Unskilled worker	1935 (16.0)	520 (15.6)	21 (19.6)	
Cohabitation of parents during pregnancy∗				<0.1%
Do not live together	>225 (>1.9)	<81 (<2.4)	< 5 (<5.0)	
Live together	<11,860 (<98.1)	>3260 (>97.6)	>103 (>95.0)	
Maternal age of menarche				0.8%
Earlier than peers	3042 (25.4)	872 (26.3)	22 (20.4)	
Same time as peers	6880 (57.4)	1878 (56.6)	72 (66.7)	
Later than peers	2066 (17.2)	569 (17.1)	14 (13.0)	
Maternal prepregnancy BMI, kg/m2				1.4%
Underweight (<18.5)	807 (6.8)	218 (6.6)	9 (8.3)	
Normal (≥18.5-<25)	7349 (61.7)	2068 (62.7)	72 (66.7)	
Overweight (≥25-<30)	2512 (21.1)	708 (21.5)	20 (18.5)	
Obese (>30)	1245 (10.5)	306 (9.3)	7 (6.5)	
Maternal smoking during pregnancy, cigarettes/d				0.3%
Nonsmoker	8594 (71.3)	2517 (75.6)	73 (67.6)	
≤10 cigarettes	2716 (22.5)	677 (20.3)	28 (25.9)	
>10	735 (6.1)	136 (4.1)	7 (6.5)	

*AD*, Atopic dermatitis; *BMI*, body mass index; *GDPR*, general data protection regulation.

∗ Missing’s are rounded up or down due to GDPR.

† Mean BMI at 7 years.

‡ Asthma, hay fever, or food allergies reported by the mother in interview 1 in Danish National Birth Cohort.

### Statistical analyses

To investigate the association between AD in childhood and pubertal development, we estimated the crude and adjusted mean monthly differences (and their 95% confidence intervals [CIs]) in age at reaching the pubertal milestones among girls and boys with AD (doctor-diagnosed and hospital-diagnosed) compared to the reference group of girls and boys without AD. The data on pubertal milestones were left, right, or interval-censored because the participants responded to the questionnaires half-yearly. Therefore, we applied a multivariable regression model for interval-censored time-to-event data, assuming normally distributed residuals fitted by maximum likelihood estimation as implemented in the *intreg* procedure in STATA 17.0 MP-Parallel Edition statistical software (Statacorp LLC).^43^

The assumption of normally distributed residuals was visually inspected by plotting the cumulative incidence functions based on the normal distribution against the nonparametric Turnbull Estimator. To inspect the assumption of independent variance, we stratified these plots on levels of the explanatory variables, and the data were consistent with these assumptions.

We included all data on puberty in one model for each sex and applied the Huber-White robust variance estimation.^44^^,^^45^ This approach accounts for multiple testing by obtaining a combined estimate for the average age difference in months when reaching all pubertal milestones between children with AD and children without AD. The model is described in more detail elsewhere.^46^

We used inverse probability weights to account for the sampling regime and potential selective nonparticipation in the Puberty Cohort. The sampling weight was derived as the inverse probability of being sampled into the Puberty Cohort, as described previously.^39^ The selection weight was estimated as the inverse probability of participation in the Puberty Cohort using a multiple logistic regression model with participation as the dependent variable and *a priori* identified covariates, including parity and AD as the explanatory variables for participation.^47^ The sampling and selection weights were multiplied, after which they were included in all models to create a pseudo-population generalizable to the source population of eligible children. To account for the clustering of siblings and the use of weights, we fitted all models with robust standard errors.

We conducted a sensitivity analysis using an algorithm developed by Benn et al^42^ that applies various questions from the DNBC to identify AD when the children were 18 months old.

In a subanalysis, we investigated persistent or recurrent AD within the last 12 months at the age of 7 years, hypothesizing that these children are disturbed during sleep and have impaired skin barrier because of active eczema in close relation to the initiation of puberty. In both sensitivity and subanalyses, we estimated selection weights for participation in the questionnaire at 18 months (the algorithm for AD) and in the 7-year follow-up (persistent or recurrent AD).

## Results

Of the 15,534 children included in this study, 3341 (21.5%) had self-reported doctor-diagnosed AD (boys accounted for 51.2%, Table I). Only 108 (0.7%) had a diagnosis in the DNPR (boys accounted for 58.3%). By the age of 7 years, around 26% of the children with self-reported doctor-diagnosed AD reported activity in their disease within the last 12 months, while 55.3% of the children with a diagnosis code reported activity in their disease at 7 years.

Mothers of children with AD more often had atopy themselves, had higher education levels, and smoked less than mothers of children without AD (Table I).

**Fig 2:**
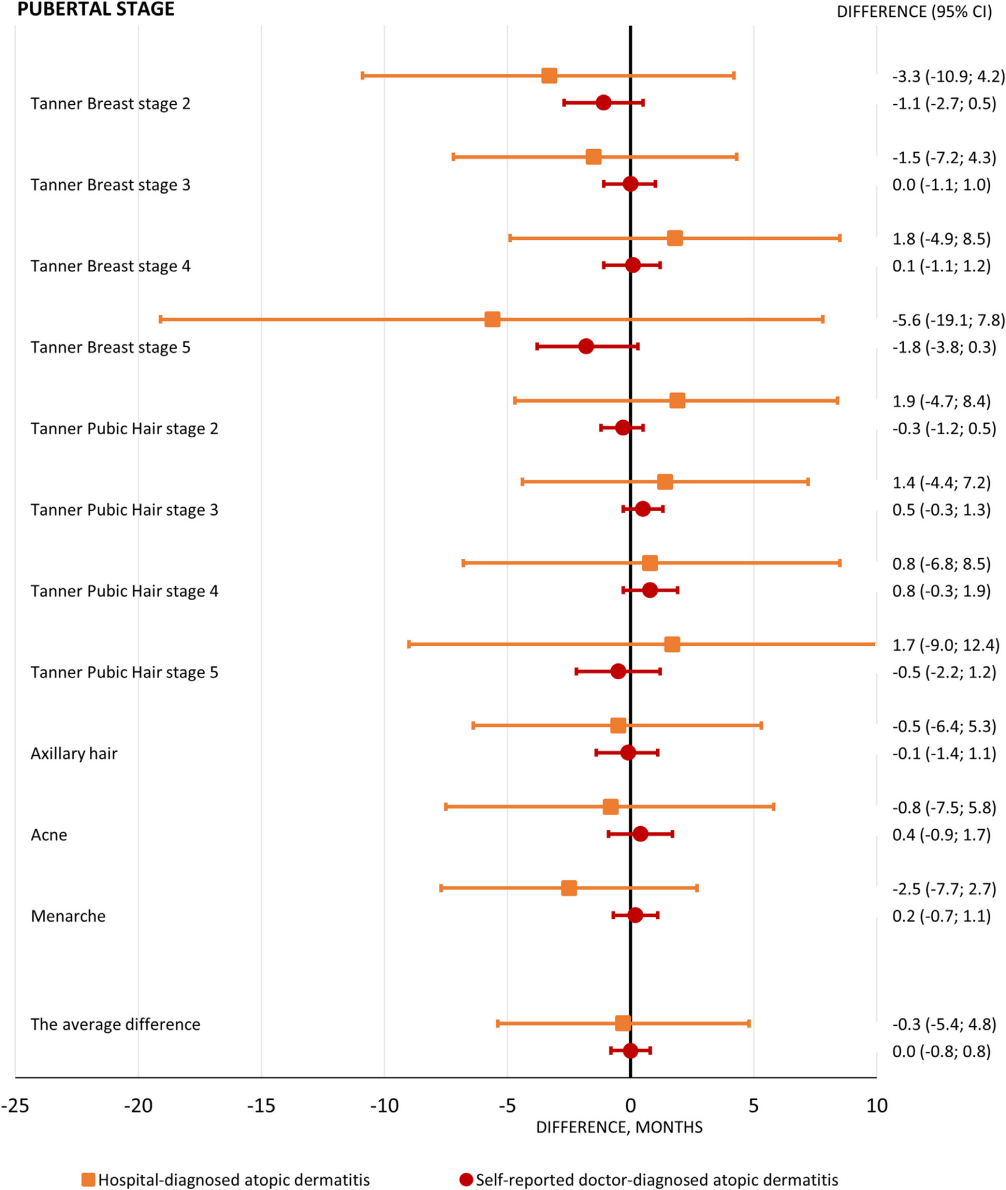
Girls. Estimated age differences in pubertal development with 95% CIs. The reference was girls without atopic dermatitis, and the analyses were adjusted for maternal age at menarche, maternal body mass index, maternal smoking during pregnancy, cohabitation of parents during pregnancy, and socioeconomic status of parents. *CIs*, Confidence intervals.

**Fig 3:**
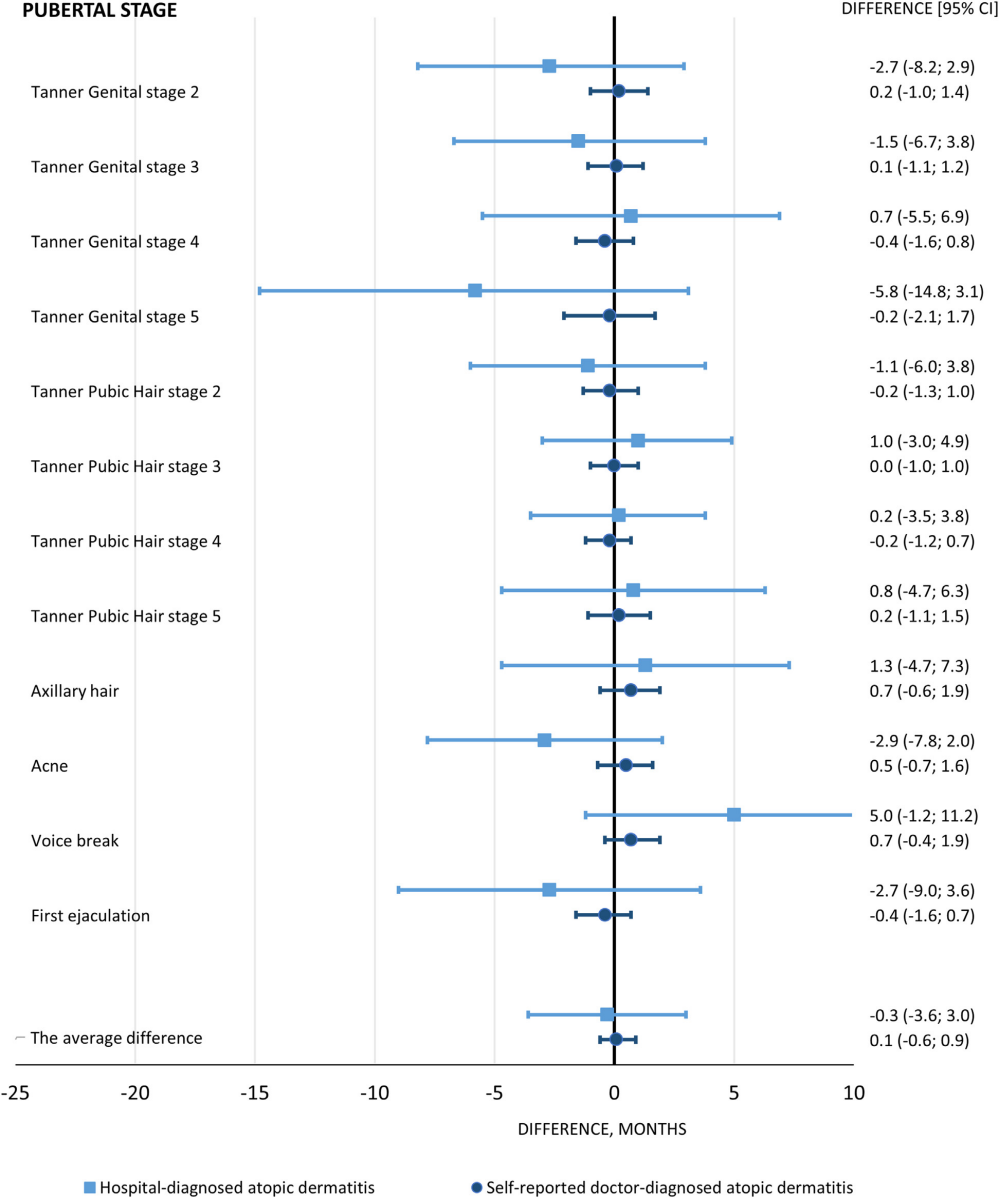
Boys. Estimated age differences in pubertal development with 95% CIs. The reference was boys without atopic dermatitis, and the analyses were adjusted for maternal age at menarche, maternal body mass index, maternal smoking during pregnancy, cohabitation of parents during pregnancy, and socioeconomic status of parents. *CIs*, Confidence intervals.

In both girls and boys, AD was associated neither with pubertal development in self-reported, doctor-diagnosed AD (the average difference for girls: 0.0 months, [95% CI: −0.8; 0.8] and for boys: 0.1 months, [95% CI: −0.6; 0.9]) nor with hospital-diagnosed AD (the average difference for girls: −0.3 months, [95% CI: −5.4; 4.8] and for boys: −0.3 months, [95% CI: −3.6; 3.0]) (Figs 2 and 3).

The results from our sensitivity analysis using an algorithm for AD were comparable with findings from the primary analysis (the average difference: girls: −0.2 months, [95% CI: −1.2; 0.9] and boys: −0.4 months, [95% CI: −1.3; 0.5]) (Supplementary Table I, available via Mendeley at https://data.mendeley.com/datasets/4jhwhhnt93/2).

We found no associations between persistent or recurrent AD at 7 years and pubertal development (Supplementary Table 2, available via Mendeley at https://data.mendeley.com/datasets/4jhwhhnt93/2).

## Discussion

In this large population-based cohort study, we observed no associations between AD and pubertal development among girls and boys. The sensitivity analysis using an algorithm to identify AD at 18 months supported the findings from the primary analysis as did the subanalysis of persistent or recurrent AD around the age of 7 years before the onset of puberty.

The present study has several strengths, including the high participation rate in the Puberty Cohort (70%), which reduces the risk of selection bias.^48^ The DNBC has been found to include more women with higher socioeconomic status^49^; which, though not necessarily biasing the estimates, might affect generalizability.^50^ We were able to study both mildly and severely affected children, which is important since approximately 80% of patients suffering from AD run a mild disease course.^51^ The hospital-diagnosed AD is less prone to recall bias than self-reported doctor-diagnosed AD; however, it includes only a selected group of AD patients. The extent to which this group is more affected by their disease than patients not included in the DNPR remains uncertain, particularly when compared with patients seen by private practice dermatologists. A study from 2021^52^ reported considerable differences regarding treatment strategies between general practitioners, private practice dermatologists, and hospital-based dermatologists in Denmark. Other factors than severity that may influence the referral to a department of dermatology are reported to be, for example, lower treatment compliance in less resourceful families or higher demands from resourceful families.^53^ We found comparable results for both groups with AD.

Hospital-diagnosed AD included only AD diagnoses the children had received as the primary reason for hospital contact until the age of 8 years. The diagnostic code in the DNPR has a positive predictive value of 98% in children (<18 years).^53^ The primary diagnosis is the main purpose for hospitalization and treatment, and it is most often given at a dermatology or pediatrics department.^53^ The use of self-reported information on AD may, on the other hand, introduce a risk of misclassification, which, though, will likely be nondifferential as the mother provided information during childhood before puberty onset. Mothers have previously been found to be quite accurate when reporting AD in their children.^54^

We did not address missing data, which ranged from <0.1% to 1.4% for covariates. Complete case analysis is acceptable when missing data are below 5% and unlikely to disproportionately affect specific patient groups, ensuring minimal impact on the results.^55^ Another limitation of the present study was the lack of treatment information. Therefore, we were unable to conduct an analysis investigating, for example, systemic versus topical treatment as an indicator of disease control and severity. We must assume that the children with AD studied here were quite heterogeneous in terms of disease severity. We tried to use a diagnostic algorithm by Benn et al^42^ where the classification of AD was validated against a dermatologist diagnosing the children at a clinical examination. This AD definition has a sensitivity of 81% and a specificity of 91%^42^; however, the algorithm could be prone to selection bias due to missing data as nearly 3000 children did not receive the questions used in the algorithm. We found comparable results with the primary analysis.

Some degree of misclassification from the self-reported pubertal development cannot be ruled out, as was found in the Puberty Cohort validation study. Nonetheless, it was concluded that these self-reported pubertal development information remained valid in large epidemiological studies.^56^

Previous research has predominantly focused on height growth and growth spurt among children with AD, except for one study that only examined puberty in girls. Most studies indicate a trend toward temporary height growth delay.^17^^,^^23^, ^24^, ^25^, ^26^, ^27^ Our study stands out due to its inclusion of several detailed markers for puberty. The results of the present study are reassuring for young patients with AD approaching puberty and reproductive health in adult life.

## Conclusion

In conclusion, we found no association between AD and pubertal development in girls and boys.

## Declaration of generative AI and AI-assisted technologies in the writing process

During the preparation of this work, the authors used ChatGPT, Grammarly and Writefull to improve language and readability. After using these tools, the authors further reviewed and edited the content as needed. They take full responsibility for the content of the publication.

## Conflicts of interest

Dr Deleuran has received research support, honoraria for lecturing and/or consulting/advisory board agreements from AbbVie, Eli Lilly, LEO Pharma, Incyte, La Roche Posay, NUMAB Therapeutics AG, Pierre Fabre, Pfizer, Regeneron Pharmaceuticals, Inc., Sanofi Genzyme, Almirall, and Kymab (not relevant to the present article). Drs Kjersgaard, Ernst, Clemmensen, Harrits Lunddorf, Arendt, Brix, Arah, and Ramlau-Hansen have no conflicts of interest to declare.
